# The ultrastructural damage caused by *Eugenia zeyheri* and *Syzygium legatii* acetone leaf extracts on pathogenic *Escherichia coli*

**DOI:** 10.1186/s12917-020-02547-5

**Published:** 2020-09-04

**Authors:** Ibukun M. Famuyide, Folorunso O. Fasina, Jacobus N. Eloff, Lyndy J. McGaw

**Affiliations:** 1 Department of Paraclinical Sciences, Phytomedicine Programme, Faculty of Veterinary Science, University of Pretoria, Private Bag X04, Onderstepoort, 0110 South Africa; 2 Dept of Veterinary Tropical Diseases, Faculty of Veterinary Science, University of Pretoria, Private Bag X04, Onderstepoort, 0110 South Africa; 3 Present Address: Emergency Centre for Transboundary Animal Diseases-Food and Agriculture Organization of the United Nations (ECTAD-FAO), House H. Sida, Ali Hassan Mwinyi Road, Ada Estate, Dar es Salaam, Tanzania

**Keywords:** Scanning Electron microscopy, Transmission Electron microscopy, *Escherichia coli*, Fluorescence microscopy, Myrtaceae, *Eugenia*, *Syzygium*

## Abstract

**Background:**

Antibiotics are commonly added to livestock feeds in sub-therapeutic doses as growth promoters and for prophylaxis against pathogenic microbes, especially those implicated in diarrhoea. While this practice has improved livestock production, it is a major cause of antimicrobial resistance in microbes affecting livestock and humans. This has led to the banning of prophylactic antibiotic use in animals in many countries. To compensate for this, alternatives have been sought from natural sources such as plants. While many studies have reported the antimicrobial activity of medicinal plants with potential for use as phytogenic/botanical feed additives, little information exists on their mode of action. This study is based on our earlier work and describes ultrastructural damage induced by acetone crude leaf extracts of *Syzygium legatii* and *Eugenia zeyheri* (Myrtaceae) active against diarrhoeagenic *E. coli* of swine origin using scanning electron microscopy (SEM), transmission electron microscopy (TEM), and fluorescent microscopy (FM). Gas chromatography/mass spectrometry (GC-MS) was used to investigate the chemical composition of plant extracts.

**Results:**

The extracts damaged the internal and external anatomy of the cytoplasmic membrane and inner structure at a concentration of 0.04 mg/mL. Extracts also led to an increased influx of propidium iodide into treated bacterial cells suggesting compromised cellular integrity and cellular damage. Non-polar compounds such as α-amyrin, friedelan-3-one, lupeol, and β-sitosterol were abundant in the extracts.

**Conclusions:**

The extracts of *S. legatii* and *E. zeyheri* caused ultrastructural damage to *E. coli* cells characterized by altered external and internal morphology. These observations may assist in elucidating the mode of action of the extracts.

## Background

Antimicrobial resistance remains a major threat to human health globally. The use of antibiotics as growth promoters in livestock feeds is a major risk for development of antibiotic resistance in humans due to the presence of residual antibiotics in edible animal products and contamination of the environment with animal faecal matter [1, 2]. Due to the strong association between antibiotic resistance and the use of antimicrobials in livestock, especially as feed additives, some countries particularly in the European Union have banned use of antibiotics as growth promoters [3]. This has motivated the search for suitable alternatives including the use of standardized plant extracts or isolated compounds. Active and safe compounds isolated from plants can additionally serve as drug leads for therapeutic purposes [4].

Molecular biology techniques and microscopy are used to study the mechanism of action of antimicrobial compounds [5]. Electron microscopy is used in biomedical research to study the ultrastructural morphology of bacterial cells [6, 7]. Scanning and transmission electron microscopy aid the visualization of images at high resolution, providing detailed information on normal or abnormal external and internal cellular morphology such as the cell membrane, cytoplasm, nucleus, organelles and cytoskeletal structures [8, 9]. These methods have greater advantages over conventional light microscopy because they are able to give three-dimensional images and higher image resolutions compared to light microscopy [10].

Although many studies have reported antibacterial activity of several plant extracts, fractions, and isolated compounds against a wide range of microbes however, information describing their ultrastructural effects are scanty.

Many *Syzygium* and *Eugenia* species have antimicrobial activity [11, 12]. The fruits and leaves of *Eugenia zeyheri* and *S. legatii* are consumed by humans and animals as food [13, 14]. In a recent study, acetone leaf crude extracts of *Eugenia zeyheri* and *Syzygium legatii* had excellent antimicrobial activity with MICs varying from 0.04 to 0.23 mg/ml on clinical and reference strains of *E. coli*. It also reduced the attachment of *E. coli* to intestinal cells in a Caco-2 cell adhesion assay [15].

This study described the morphological and ultrastructural alterations caused by crude acetone extracts of *S. legatii* and *E. zeyheri* on a diarrhoeagenic *E. coli* strain of swine origin, using scanning and transmission electron microscopy. The effect on membrane permeability of the bacterial cells upon treatment with the extracts was also investigated with propidium iodide which is an intact membrane-impermeable fluorescent dye [16]. Gas chromatography coupled with mass spectrometry (GC-MS) was used to investigate compounds present in the plant extracts.

## Results

### Electron microscopy

**Fig. 1:**
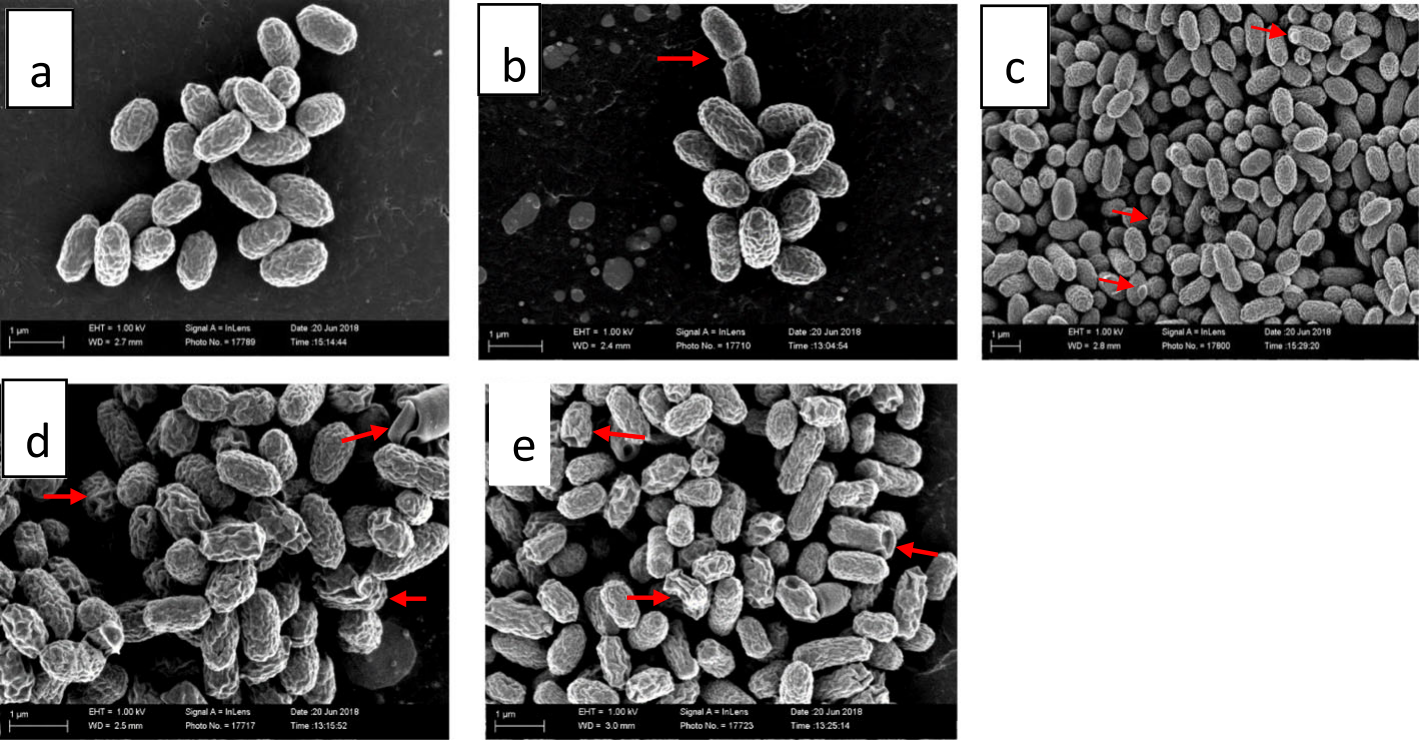
Scanning electron microscopic morphology of: **a** = untreated *E. coli* cells (negative control) after 24 h (cells are clustered and have normal short rod shape and size.), **b** = solvent control after 24 h (cells are clustered and have normal short rod shape and size), **c** = *E. coli* cells after 24 h exposure to 1 μg/mL gentamicin (many of the cells are normal with a few cells having rough or wrinkled surfaces and protrusions), **d** = *E. coli* cells after 3 h exposure to 0.04 mg/mL extract of *Syzygium legatii* (cells have rough or wrinkled surfaces, altered shape, cavitations and cracks), **e** = *E. coli* cells after 3-h exposure to extract of *Eugenia zeyheri* (cells are wrinkled, shrunken and rough with cavitations)

**Fig. 2:**
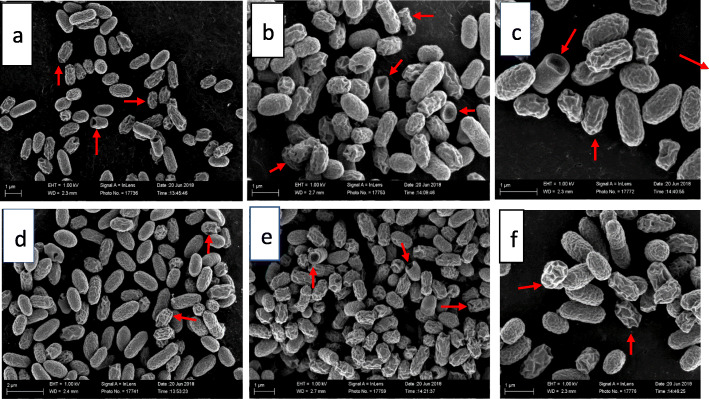
Scanning electron microscopic morphology of *E. coli* cells: **a** = after 6 h exposure to 0.04 mg/mL extract of *Syzygium legatii* (cells have rough or wrinkled surfaces, altered shape, and telescoping or invagination of some cells), **b** = after 12 h exposure to 0.04 mg/mL extract of *Syzygium legatii* (cells have rough surfaces, altered shape, and telescoping or invagination of some cells, protrusions on the surface), **c** = after 24 h exposure to 0.04 mg/mL extract of *Syzygium legatii* (cells are wrinkled, shrunken and rough with surface protrusions), **d** = after 6-h exposure to 0.04 mg/mL extract of *Eugenia zeyheri* (cells appear rough, twisted and wrinkled), **e** = after 12-h exposure to 0.04 mg/mL extract of *Eugenia zeyheri* (cells are wrinkled, shrunken, rough with cavitation, projections on the cell surface), **f** = after 24-h exposure to 0.04 mg/mL extract of *Eugenia zeyheri* (cells are wrinkled, shrunken, and rough

There were significant external ultrastructural changes in the scanning electron microscopic investigation of treated cells compared to the control cells as early as the third hour of treatment of bacteria with both plant extracts (Fig. 1d, e and 2a-f). Many of the cell populations in both extract-treated bacteria appeared rough, twisted, wrinkled and misshapen. Some cells were telescoping or had invagination, while others had protrusions on their surfaces. These effects were observed after 6, 12 and 24 h of exposure of the bacteria to both plant extracts.

**Fig. 3:**
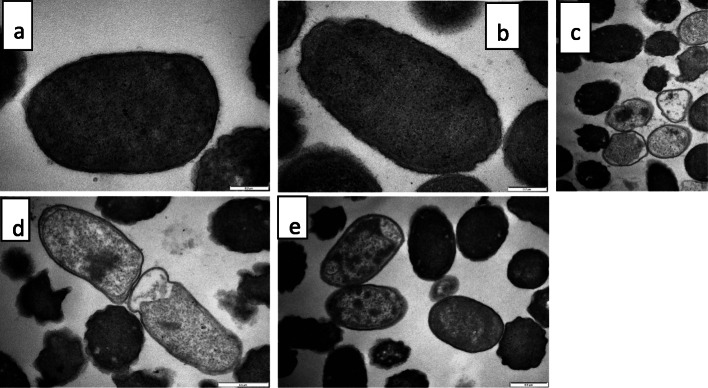
Transmission electron microscopic morphology of *E. coli* cells: **a** = untreated control after 24 h (cell have uniform cytoplasmic density and intact cell membranes), **b** = acetone solvent control after 24 h (cells have uniform cytoplasmic density, intact cell membrane), **c** = gentamicin-treated cells after 24 h (some cells are electron sparse, and detached from the cell wall), **d** = after 3-h exposure to 0.04 mg/mL extract of *Syzygium legatii* (cell membrane detaching from cell, translucent cytoplasm, shrunken, misshapen cells), **e** = after 3-h exposure to 0.04 mg/mL extract of *Eugenia zeyheri* (cell membrane detaching from cell, with islands of condensed chromatin)

**Fig. 4:**
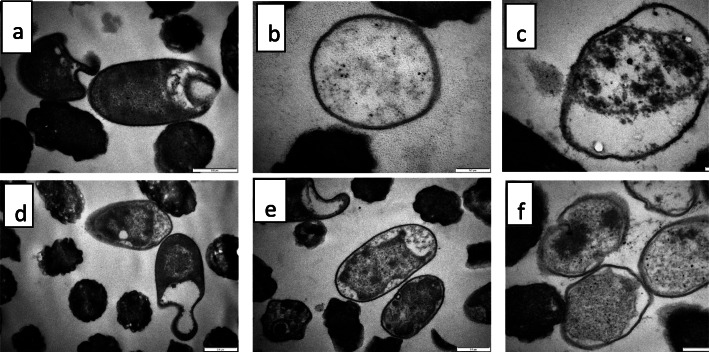
Transmission electron microscopic morphology of *E. coli* cells: **a** = after 6-h exposure to 0.04 mg/mL extract of *Syzygium legatii* (cell membrane detaching from cell, misshapen cells), **b** = after 12-h exposure to 0.04 mg/mL extract of *Syzygium legatii* (late stage cell death. Ghost cells with no cytoplasmic content. Cell walls appear intact), **c** = after 24-h exposure to 0.04 mg/mL extract of *Syzygium legatii* (cell at late stage of death, condensed chromatin), **d** = after 6-h exposure to 0.04 mg/mL extract of *Eugenia zeyheri* (cell membrane detaching from cell, misshapen cells with loss of structure), **e** = after 12-h exposure to 0.04 mg/mL extract of *Eugenia zeyheri* (cell membrane detaching from cell, misshapen cells with loss of turgidity, condensation and aggregation of nuclear chromatin), **f** = after 24-h exposure to 0.04 mg/mL extract of *Eugenia zeyheri (c*ells show detachment of membrane from cell wall. Electron sparse cytoplasm, condensed chromatin)

Observations from transmission electron microscopy (Fig. 3a-e and 4a-f) were also similar. Alterations in the cells were observed as early as the 3rd h of extract treatment (Fig. 3d and d), progressing till the 24th h of treatment. Some cells had chromatin condensation which was packed into apoptotic-like bodies (e.g. Figure 3d, e; Fig. 4e, f) while the membrane of some cells was detached from the cell wall with electron sparse cytoplasm (e.g. Figure 4c, and d).

### Fluorescent microscopy

**Fig. 5:**
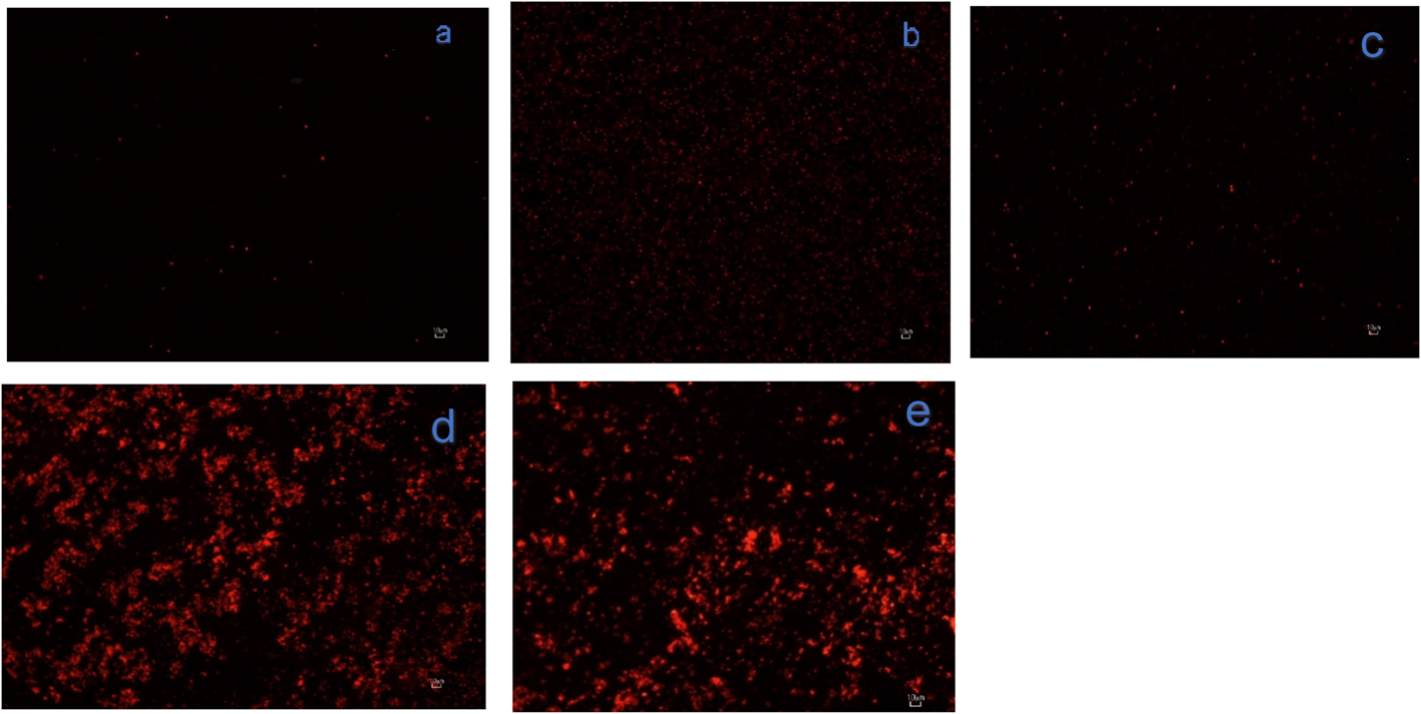
Fluorescence microscopy images of *E. coli* stained with propidium iodide: **a** negative control (untreated), **b** negative control (heat -killed), **c** gentamicin (1 μg/mL) treated for 3 h, **d** *S. legatii* (0.04 mg/mL) treated for 3 h, e) *E. zeyheri* (0.04 mg/mL) treated for 3 h. The red fluorescence indicates cells with damaged membrane

Fluorescent microscopic results revealed an increased entry of the fluorescent dye into extract-treated bacterial cells, seen by the amount of fluorescence in treated cells compared to the untreated control (Fig. 5d and e). A small amount of red fluorescence was observed in the untreated control (Fig. 5a) which showed that most of the bacterial cells were viable and the fluorescence observed may be due to small bacteria that died naturally, while more fluorescence was observed in heat-killed bacteria (Fig. 5b).

### Gas chromatography-mass spectroscopy analysis of plant extracts

The gas chromatographic-mass spectrometric analysis of acetone leaf extracts of *E. zeyheri* (Table 1) and *S. legatii* (Table 2) revealed the presence of compounds such as terpenes, steroids, alkane hydrocarbons, epoxides, and saturated fatty acids. Lupeol and α-Amyrin were most abundant in *E. zeyheri* while friedelan-3-one was most abundant in *S. legatii*.

**Table 1:** Major compounds detected in the acetone crude leaf extract of *Eugenia zeyheri* by GC-MS

S/N	Compound	RT (s)	Molecular formula	MW	% Peak Area	Similarity index (%)
1	α-Amyrin	1684.7	C_30_H_50_O	426	23.272	88.9
2	Lupeol	1687.1	C_30_H_50_O	426	24.165	89.4
3	γ-Sitosterol	1658.2	C_29_H_50_O	414	0.22951	62.8
4	2,​6,​10-​Dodecatriene, 1-​bromo-​3,​7,​11-​trimethyl-	1654	C_15_H_25_Br	284	1.6429	79.9
5	Olean-12-en-3β-ol (6CI,8CI)	1663.1	C_30_H_50_O	426	4.9815	89.2
6	Decane	133.3	C_10_H_22_	142	0.3568	95.2
7	trisiloxane, 1,1,1,5,5,5-hexamethyl-3-[(trimethylsilyl)oxy]-	134.6	C_9_H_28_O_3_Si_4_	296	0.3568	75.2
8	Dodecane	315.8	C_12_H_26_	170	0.44363	94.4
9	Tetradecane	505.4	C_14_H_30_	198	0.45213	96.1
10	Diethyl phthalate	632.2	C_12_H_14_O_4_	222	0.042262	94.1
11	Hexadecane	679.2	C_16_H_34_	226	0.28988	94.6
12	n-Hexadecanoic acid	959.8	C_16_H_32_O_2_	256	0.47217	91.9
13	Heneicosane	1112.5	C_21_H_44_	296	0.45644	93.2
14	Squalene	1450.9	C_30_H_50_	410	0.21611	92.1
15	Dl-α-Tocopherol	1592.5	C_29_H_50_O_2_	430	0.68369	88.2

**Table 2:** Major compounds detected in the acetone crude leaf extract of *Syzygium legatii* by GC-MS

S/N	Compound	RT (s)	Molecular formula	MW	% Peak Area	Similarity index (%)
1	Friedelan-3-one	1734.2	C_3_0H_50_O	426	4.6496	88.8
2	Oleanolic acid	1842	C_3_0H_48_O_3_	456	1.3333	80.4
3	Urs-12-en-28-ol	1814.1	C_3_0H_50_O	426	1.17	84.5
4	α-Amyrin	1680.5	C_3_0H_50_O	426	1.2047	88.8
5	Dl-α-Tocopherol	1592.4	C_29_H_50_O_2_	430	1.4543	89.5
6	3,7,11,15-Tetramethyl-2-hexadecen-1-ol	892.6	C_20_H_40_O	296	1.1379	89.3
7	Neophytadiene	862.3	C_20_H_38_	278	1.5963	89.7
8	3-Eicosene, (E)-	800.9	C_20_H_40_	280	1.2195	81.1
9	Hexadecane	679.5	C_16_H_34_	226	1.6552	94.3
10	Caryophyllene oxide	642.4	C_15_H_24_O	220	1.8127	89
11	4H-Imidazol-4-one, 2-amino-1,5-dihydro-	614.7	C_3_H_5_N_3_O	99	1.3649	80.4
12	Tetradecane	505.9	C_14_H_30_	198	2.649	94.3
13	Dodecane	316.6	C_12_H_26_	170	2.5936	94.2
14	Ethanedicarboxamide, N-(3,5-dimethylphenyl)-N′-[2-(1-piperazinyl)ethyl]-	317.9	C_16_H_24_N_4_O_2_	304	2.628	92.4
15	Decane	135.6	C_10_H_22_	142	1.8075	96

*RT* Retention time, *MW* Molecular weight.

*RT* Retention time, *MW* Molecular weight.

## Discussion

Microscopic techniques have long been used to assess changes in cells. The changes in the bacterial morphology were determined by scanning (SEM) and transmission electron microscopy (TEM) at different times of exposure while cellular integrity after a 3 h treatment with extracts was determined by fluorescence microscopy. SEM provides insight on the effects of antibacterial agents on external morphology and surface characteristics of bacterial cells, while TEM indicates the internal architecture of normal and abnormal cells. The two techniques combined can give a useful understanding of the antibacterial mechanism of action of novel antibacterial agents [8]. The negative control (acetone) had no effect on the ultrastructure, but the positive control (gentamicin) caused many of the bacterial cells to have roughened or wrinkled surfaces with some having protrusions.

The appearance of some cells with chromatin condensation packed into apoptotic-like bodies are hallmarks of cellular apoptosis [17]. It can be inferred that many of the treated bacterial cells by the plant extracts may be undergoing apoptosis. Therefore, the mode of action of the plant extracts may be by causing apoptosis directly or indirectly.

The changes are similar to those observed in previous studies with silver ion [5], polymyxin-B and miconazole [18], as well as honey [19] on *E. coli*. In a related study, exposure of pathogenic *E. coli* to cranberry extracts caused morphological damage such as cellular deformation, breakage of cell wall and membrane, condensation of cellular material, and presence of large amounts of cytoplasmic material and membrane debris in the cell’s surrounding environment [20]. These results, which are like our findings, showed that the plant extracts induced pathological damage to *E. coli* possibly through primary effects on metabolism that led to the alteration of cellular structures. In one study, treatment of *Bacillus cereus* and *Campylobacter jejuni* with the ethanolic extract of *Annona squamosa* caused alterations and distortions in bacterial cell shape, abnormal elongations, leakage of intracellular contents, and depletion of the cytoplasm [8]. In another study, treatment of *E. coli* with the ethanol extract of clove buds (*Syzygium aromaticum*) caused loss of regular cellular shapes and cell membrane disintegration from the cell wall [21]. Treatment of *E. coli* with cinnamaldehyde caused the separation of cytoplasmic membranes from the cell wall, cell wall and cell membrane lysis, cytoplasmic content leakage, cytoplasmic content polarization, and cell distortion [22]. These findings are similar to those of the present study. The outcome of this study may therefore provide further insights into innovative alternative methods of bacterial management through disruption of the pathogen integrity.

The integrity of the bacterial membrane following exposure to *E. zeyheri* and *S. legatii* was assessed using a fluorescent dye, propidium iodide. This dye is known to intercalate with bases of deoxyribonucleic acid (DNA) to fluoresce. Propidium iodide can enter only a compromised bacterial cell membrane to bind with DNA as an intact cell membrane will exclude it.

Increased fluorescence was detected in gentamicin-treated cells, indicating that it also affected the cell membrane. The primary effect of gentamicin on bacteria is to bind on the 30S subunit of bacterial ribosome, the structural damage observed is therefore a secondary effect. The high fluorescence observed from treatment of bacteria with the extract of *E. zeyheri* and *S. legatii* indicated damage to the integrity of the bacterial cell membrane. In a similar study, the aqueous extract of *Cassia alata* caused a high number of cell deaths in *Streptococcus epidermidis* and *Pseudomonas aeruginosa* based on fluorescence microscopy [23].

The fluorescence results support the observations in the electron microscopy study. Cell abnormalities observed in the electron microscopy results such as abnormal cellular integrity, and/or partial or complete loss of cytoplasmic contents may be responsible for the highly permeable state of the cells to PI. It appeared that the compounds present in the extracts were able to penetrate the peptidoglycan layer of the bacterial cell into the cell membrane to exert the antibacterial effects. Possible targets for the compounds may be present on this layer [24]. More studies are needed to confirm these observations. In a previous study, lupeol, isolated from *Curtisia dentata* leaves had antibacterial activity against *E. coli* (ATCC 25922) with an MIC of 250 μg/mL [25]. In another report, α-amyrin had an MIC of 64 μg/mL against *Staphylococcus aureus* (ATCC 43300) [26]. It may be worthwhile to isolate and characterize the bioactive compounds from the plant extracts from this study and determine their antibacterial activity and safety which may serve as new antibacterial drugs.

## Conclusion

The extracts of *S. legatii* and *E. zeyheri* caused morphological and ultrastructural damage to *E. coli* when exposed to inhibitory concentrations of the extracts. These effects were characterized by disruptions and disfigurations in the cell shape and surface cell wall formation. The extracts affected the membrane integrity of the bacterial cells shown by increased fluorescence. We conclude that the plant extracts have potential for therapeutic use. Further work may include exploring the molecular mechanisms and precise cellular target(s) responsible for the effects seen in this study.

## Methods

### Collection of plant material, drying and storage

After obtaining the necessary permit, leaves of two selected plants namely *Eugenia zeyher*i (Harv.) Harv. and *Syzygium legatii* Burtt Davy & Greenway were harvested in April 2017, at the Lowveld National Botanical Garden in Nelspruit, Mpumalanga, South Africa. Herbarium specimens were prepared and deposited in the HGWJ Schweickerdt Herbarium of the University of Pretoria for authentication while herbarium specimen identity number (PRU) were obtained. These were 123,617 and 123,619 for *E. zeyheri* and *S. legatii* respectively. The leaves were placed in open mesh loosely woven bags and dried under room temperature with adequate ventilation. Using a Janke and Künkel Model A10 mill, the dried leaves were ground to a fine powder, weighed and stored in at room temperature in closed jam jars [27].

### Extraction

Two grams of dried powdered plant material was extracted with 20 mL of acetone technical grade, Merck) in 50 mL centrifuge tubes. Acetone is widely considered a solvent of choice because it can extract compounds with a wide range of polarities, it is relatively easy to remove from extracts and it is non-toxic to bioassays systems [28]. The tube containing the mixture was vigorously shaken and sonicated for 20 min followed by centrifugation for 10 min at 4000 X g. The supernatant was then filtered using a Whatman No. 1 filter paper into a pre-weighed glass container and then dried under a cold stream of air in a fume hood at room temperature to obtain a dried extract. The dried extracts for were dissolved in the required volume of acetone for the bioassays.

### Bacterial strain

An enterotoxigenic *E. coli* (possessing the STA and F6 virulence genes) isolated from a diarrhoeic piglet was obtained from the Department of Veterinary Tropical Diseases, Faculty of Veterinary Sciences, University of Pretoria. The organism was maintained on Tryptic Soy Agar (TSA, Oxoid) at 4 °C.

### Preparation of *E. coli* culture for electron microscopy

*Escherichia coli* was grown in TSA for 18 h after which a single colony was inoculated into Tryptic Soy Broth aseptically and incubated at 37 °C on a shaker for 18 h. After this, an inoculum equivalent to a McFarland No 1 standard (3.6 × 10^8^ cfu/mL) was prepared from the 18 h culture. Prior to this, TSA was prepared, and 5 mL of the molten agar was gently poured into 35 mm diameter sterile tissue culture plates to form a smooth and evenly spread surface and allowed to solidify in a sterile environment. The 35 mm agar plates were inoculated with the appropriately adjusted *E. coli* suspension which were spread evenly on the agar surface with a sterile glass spreader. The plates were then incubated at 37 °C for 12 h under aerobic conditions. The plates were divided into two groups. Plates in group one was flooded with 100 μl of *E. zeyheri* extract while those in group two were flooded with the extract of *S. legatii*, both at 0.04 mg/mL in acetone. This concentration represented the minimum inhibitory concentration of the extracts on the test bacterium in our previous study. One plate was flooded with 100 μl of 50% acetone to represent the solvent control. One plate also served as the untreated control, while another was treated with 100 μl gentamicin (1 μg/mL) as positive control. The plates were then incubated under aerobic conditions at 37 °C. After 0, 3, 6, 12, and 24 h, separate plates were removed from the incubator and flooded with 1 mL of 2.5% glutaraldehyde in 0.075 M phosphate buffer solution (pH 7.4) to fix the samples for 60 min. The bacterial biofilms were then collected from each plate using sterile loops and transferred into 2 mL microcentrifuge tubes containing 1.5 mL of 0.5% glutaraldehyde in order to fix the cells for 1 h. Glutaraldehyde was removed with a pipette and the cells were washed thrice with 0.075 M sodium buffer for 10 min each. The samples were then fixed with osmium tetroxide (OsO_4_, Merck, Darmstadt, Germany) in a fume hood for 30 min. Osmium tetroxide was removed and the cells were rinsed three times with the sodium phosphate buffer. Dehydration was done with increasing ethanol concentrations of 50, 70, 90 and 100% for 15 min each. The 100% ethanol step was repeated three times before preparation for scanning and transmission electron microscopy.

### Scanning electron microscopy (SEM) sample processing

Following the last 100% ethanol dehydration from the step above, hexamethyldisilazane (HMDS) was added at 50% in ethanol for 30 min. Hexamethyldisilazane/ethanol was replaced with two changes of pure HMDS for 1 h each. A small droplet (0.05 mL) containing sample was placed on highly polished carbon discs and left open in a fume hood to dry overnight. These carbon discs were stuck using double-sided carbon tape onto aluminium stubs. Samples were made conductive by exposure to ruthenium tetroxide (RuO_4_) for 45 min [29]. Samples were then viewed with a Zeiss Ultra Plus Field Emission Gun Scanning Electron Microscope (FEGSEM) at the Electron Microscope Unit of the University of Pretoria.

### Transmission electron microscopy (TEM) sample processing

After the third dehydration step in 100%, ethanol was removed and replaced with propylene for 2 h. The propylene oxide was replaced with an Epon type epoxy resin (TAAB 812) and infiltrated for 5 h. Pellets were removed from the microcentrifuge tubes, placed in embedding moulds, and polymerised for 48 h. After this, sections were made with a Reichert Ultracut E ultramicrotome using a diamond knife and picked up onto copper grids. The sections on the grid were stained with 2% uranyl acetate followed by 2 min staining in Reynold’s lead citrate. Sections were viewed and photographed with a Philips EM 10 transmission electron microscope (Eindhoven, Netherlands) in the Electron Microscopy Unit, Department of Anatomy and Physiology, Faculty of Veterinary Sciences, University of Pretoria.

### Propidium iodide (PI) uptake assay: cell viability assessment

This was done as previously reported [30] with slight modifications. Briefly, the *E. coli* cells were grown in TSB for 18 h, washed twice with phosphate buffer saline (PBS) and then adjusted to 10^6^ CFU/mL with PBS. The cells were then incubated at 37 °C with the extracts of *E. zeyheri* and *S. legatii* at the MIC concentrations (0.04 mg/mL respectively) for 3 h. Gentamicin (1 μg/mL) served as positive control, untreated cells served as untreated negative control while heat-killed (70 °C for 60 min) bacteria served as treated negative control. Following incubation, cells were washed twice with PBS and fixed with 3.7% paraformaldehyde in 0.1 M cacodylate buffer for 20 min at room temperature. Cells were then washed twice with PBS and incubated with PI at 37 °C for 30 min in the dark. Cells were spread on a glass slide, covered by a cover slip and observed using a Nikon Eclipse TS 100-U inverted fluorescence microscope (Nikon, Champigny sur Marne, France) fitted with a Nikon Intensilight C-HGFI lamp unit at excitation and emission wavelengths of 536 and 617 nm respectively. Images were captured at 20 x magnification.

### Gas chromatography-mass spectrometry (GC-MS) of plant extracts

Analysis of chemical constituents of plant extracts of *S. legatii* and *E. zeyheri* were carried out in a LECO Pegasus 4D GC-TOFMS (LECO Africa (Pty) Ltd., Kempton Park, South Africa) on an apolar Rxi-5SilMS 30 m × 0.25 mm ID × 0.2 μm film thickness (Restek, Bellefonte, PA, USA) gas chromatography capillary column. Compound spectra were detected by electron ionization system (70 eV). Ultra-high purity grade carrier gas, Pure helium gas (Afrox, South Africa) was set at a constant flow rate of 1 mL/min. Oven temperature was held for 3 min with 5 min solvent delay programmed at 40 °C and held isothermally at 300 °C for 5 min. A 1 μL of acetone solution of the sample was injected in a splitless mode (splitless time 30s) with the injector temperature at 250 °C. Ion source temperature was maintained at 280 °C. A scan interval of 0.5 s and fragments from 40 to 550 Da was maintained. Relative quantity of the compounds in extracts was expressed as a percentage based on the peak area produced in the chromatogram. Tentative identification of the bioactive constituents was done by comparison of retention times with standard samples and by matching the spectral fragmentation patterns against commercial library mass spectra. Analysis was done at the Department of Chemistry, University of Pretoria, South Africa.

## Data Availability

The datasets used and/or analysed during the current study are available from the corresponding author on reasonable request. The plant material collected from the botanical gardens is available from the corresponding author on reasonable request.

## Abbreviations

STA: Heat-stable enterotoxin gene A
F6: Fimbrial adhesin subunit 6
PI: Propidium iodide
SEM: Scanning Electron Microscopy
TEM: Transmission Electron Microscopy
